# Association between adherence to the American Cancer Society Nutrition and Physical Activity Guidelines and stool frequency among colon cancer survivors: a cohort study

**DOI:** 10.1007/s11764-022-01288-8

**Published:** 2022-11-05

**Authors:** Anya L. Greenberg, Irina V. Tolstykh, Katherine Van Loon, Angela Laffan, Dalila Stanfield, Paige Steiding, Stacey A. Kenfield, June M. Chan, Chloe E. Atreya, Sorbarikor Piawah, Wesley Kidder, Alan P. Venook, Erin L. Van Blarigan, Madhulika G. Varma

**Affiliations:** 1 Department of Surgery, University of California, San Francisco, San Francisco, CA USA; 2 Department of Epidemiology and Biostatistics, University of California, San Francisco, San Francisco, CA USA; 3 Division of Hematology/Oncology, Department of Medicine, University of California, San Francisco, San Francisco, CA USA; 4 Helen Diller Family Comprehensive Cancer Center, University of California, San Francisco, San Francisco, CA USA; 5 Department of Urology, University of California, San Francisco, San Francisco, CA USA

**Keywords:** Nutrition, Physical activity, Health behavior, Cancer survivors, Bowel function

## Abstract

**Purpose:**

We sought to determine whether adherence to the American Cancer Society (ACS) Nutrition and Physical Activity Guidelines was associated with better bowel function among colon cancer survivors.

**Methods:**

This prospective cohort study included patients surgically treated for stage I–IV colon cancer enrolled in the Lifestyle and Outcomes after Gastrointestinal Cancer (LOGIC) study between February 2017 and May 2021. Participants were assigned an ACS score (0–6 points) at enrollment. Stool frequency (SF) was assessed every 6 months using the EORTC QLQ-CR29. Higher SF is an indication of bowel function impairment. ACS score at enrollment was examined in relation to SF at enrollment and over a 3-year period. Secondarily, we examined associations between the ACS score components (body mass index, dietary factors, and physical activity) and SF. Multivariable models were adjusted for demographic and surgical characteristics.

**Results:**

A total of 112 people with colon cancer (59% women, mean age 59.5 years) were included. Cross-sectionally, for every point increase in ACS score at enrollment, the odds of having frequent stools at enrollment decreased by 43% (CI 0.42–0.79; *p* < 0.01). Findings were similar when we examined SF as an ordinal variable and change in SF over a 3-year period. Lower consumption of red/processed meats and consuming a higher number of unique fruits and vegetables were associated with lower SF (better bowel function) at enrollment.

**Conclusions:**

Colon cancer survivors who more closely followed the ACS nutrition and physical activity guidelines had lower SF, an indication of better bowel function.

**Implications for Cancer Survivors:**

Our findings highlight the value of interventions that support health behavior modification as part of survivorship care for long-term colon cancer survivors.

**Supplementary Information:**

The online version contains supplementary material available at 10.1007/s11764-022-01288-8.

## Introduction

Excluding non-melanoma skin cancers, colorectal cancer is the third most common cancer diagnosed in both men and women in the United States (US) [1]. Over 1 million affected individuals are living in the US [2, 3], with nearly 150,000 new diagnoses annually [3]. We defined these individuals as cancer survivors from time of diagnosis, thorough the balance of their lives [4]. Due to ongoing improvements in screening, treatment, and surveillance, survival for patients diagnosed with colorectal cancer has improved [5–8]; between 1975 and 2011, the 5-year survival rate increased from 50 to 66% [5]. Adherence to the American Cancer Society Nutrition and Physical Activity Guidelines for Cancer Survivors [9] (herein referred to as the ACS guidelines) is independently associated with longer survival after colon and rectal cancer diagnosis [10, 11]. For example, a study of 1,425 patients with stage I–III colorectal cancer observed a 15% lower risk of all-cause mortality for every 1-standard deviation (SD) increase in the ACS guideline score [10]. Another study of 992 patients with stage III colon cancer showed that absolute 5-year survival probability was 9% higher for patients who adhere to the ACS guidelines than for those who do not [11]. As survival continues to improve through the emergence of new technologies and identification of best practices, understanding factors that impact patient quality of life (QoL) across time is increasingly critical to deliver patient-centered survivorship care.

Among colorectal cancer survivors, QoL has been linked to an array of physical, psychological, and social factors [12, 13]. Poor bowel function is one such factor that has been associated with worse QoL [14–17]. While a symptom-based scoring system for bowel function, the Low Anterior Resection Syndrome (LARS) Score, has been developed and validated for patients with rectal cancer [18], there is no currently validated bowel function score for use after surgery for colon cancer. The most frequently used measure of bowel function for this group is stool frequency [19]. Notably, high stool frequency, which is a component of the LARS score [14, 17], has been independently shown to correlate with worse QoL among multiple patient groups [14, 20, 21].

The association between high stool frequency and tumor location [17], surgical technique [22], and other factors outside the patient’s control have been established for patients with colon cancer. However, little is known about the impact of modifiable factors (such as physical activity, diet, and body size) on stool frequency in this group. In this prospective cohort study, we sought to determine whether adherence to the ACS guidelines was associated with lower stool frequency (better bowel function) among colon cancer survivors.

## Methods

### Study population

This prospective cohort study included adult patients diagnosed with stage I–IV colon cancer who had surgery and were enrolled in the open Lifestyle and Outcomes after Gastrointestinal Cancer (LOGIC) cohort study between February 2017 and May 2021. Adult patients receiving care at the University of California, San Francisco (UCSF) with a prior gastrointestinal cancer diagnosis are eligible to participate in the LOGIC study. Patients referred to the UCSF Gastrointestinal (GI) Oncology Survivorship Clinic receive an invitation to the study. Additionally, starting in 2020, adults with a diagnosis of colon, rectal, or anal cancer in their medical records are invited through a secure message through their electronic medical record (EMR) patient portal or paper letter (to individuals without EMR patient portal accounts).

Patients report demographic (e.g., age, gender, race, ethnicity, height), social (e.g., living arrangement), health behaviors (i.e., physical activity, weight, and diet), and QoL (including bowel function) data via online questionnaires at enrollment. Clinical, surgical, and tumor characteristics are obtained from review of patients’ medical records by the study team. Subsequently, online QoL surveys are administered up to every 6 months. Changes in patient clinical status (e.g., local recurrence, new metastasis) are obtained from review of patient medical records every 12 months by the study team.

As of December 1, 2021, 147 patients with colon cancer had consented and completed at least one online survey for LOGIC. For our study, we excluded individuals with missing physical activity, weight, or diet data (*n* = 6) and those with missing information on their stool frequency (*n* = 19) at enrollment. Additionally, we excluded individuals receiving chemotherapy at the time of enrollment (*n* = 3), those who had received radiation therapy (*n* = 3), and those diagnosed with other gastrointestinal (i.e., non-colon) cancers in addition to their colon cancer prior to enrollment (*n* = 4), since active treatment and history of other gastrointestinal cancers (and their treatment) may impact bowel function. We included 2 individuals who were treated for metachronous colon cancer; for these patients, most recent treatment history was used for our analysis. After exclusions, 112 patients were eligible for our study. Patients who had new metastasis (*n* = 3) or a local recurrence (*n* = 1) during follow-up were censored at the time of re-initiation of treatment.

The study was approved by the UCSF Institutional Review Board. All study participants signed an informed consent statement in accordance with federal and institutional guidelines.

### ACS guideline score

We quantified the degree of concordance between patients’ health behaviors and the ACS guidelines for cancer survivors at enrollment using a score developed by McCullough et al. (Appendix 1) [23]. The score was initially developed based on the 2006 ACS guidelines for cancer prevention and includes body mass index (BMI), physical activity, diet (i.e., intake of vegetables and fruits, proportion of total grains consumed that are whole grains, and intake of red and processed meat), and alcohol consumption [23–25]. It was adapted for the 2006 ACS guidelines for cancer survivors to exclude alcohol consumption, because this recommendation was not included in the guidelines for cancer survivors [26].

For our primary analysis, we used the ACS guideline score for cancer survivors, which ranges from 0 to 6, with higher scores indicating behaviors more consistent with the guidelines. This score was used as a continuous variable in our analysis.

We also quantified the degree of concordance between patients’ health behaviors and the ACS guidelines for cancer prevention [23], which includes the same components as the score for cancer survivors plus alcohol consumption. The ACS guideline score for cancer prevention ranges from 0 to 8. This score was also used as a continuous variable in our analysis.

### Body mass index

BMI was calculated using participant-reported weight and height at enrollment. Participant-reported weight in pounds was converted to kilograms and participant reported height in inches was converted to meters before calculating BMI (kg/m^2^).

### Physical activity

At enrollment, participants complete a physical activity questionnaire, which was previously validated in random samples drawn from 51,529 male health professionals ages 40–75 (Health Professionals Follow-up Study) [27] and 116,680 female registered nurses ages 25–42 (Nurses Health Study II) [28]. Briefly, participants are asked to report average time spent per week over the past year performing common recreational activities, including walking; jogging; running; bicycling; playing tennis, squash, or racquetball; swimming; participating in other aerobic exercise, lower-intensity exercise (yoga, stretching, toning), and other vigorous activities; and weight-training. Ten response options range from 0 to 11 or more hours per week for each activity. To calculate total metabolic equivalent task (MET) hours per week (MET-h/week) of physical activity, we assigned each activity a MET value, multiplied the activity-specific MET value by the amount of time the participant engaged in that activity, and summed across all activities [29].

### Dietary factors

Diet is assessed using a validated food frequency questionnaire (FFQ) that queries average weekly intake of approximately 150 items over the past year in up to 9 frequency options ranging from never or less than once per month to 6 or more times per day, as previously described [30–32]. The FFQ is administered at enrollment. For the ACS guidelines, items of interest include total servings/day and number of unique fruits and vegetables per month; the percent of total grains consumed that are whole grains; and total intake of red and processed meats. Patients report their alcohol consumption over the past year in up to 9 frequency options ranging from never or less than once per month to 6 or more times per day [30–32].

### Outcome assessment

Our primary outcome variable for this analysis was the stool frequency subscale of the 29-item European Organization for Research and Treatment of Cancer Quality of Life Core Questionnaire for colorectal cancer patients (EORTC QLQ-29) [33]. We elected to use stool frequency as a measure of bowel function to be consistent with past literature among patients diagnosed with colon cancer; there is no validated bowel function score in this population [19]. Participants have the opportunity to complete the EORTC QLQ-29 every 6 months. Up to 36 months of stool frequency data was used for repeated measures analyses.

The stool frequency subscale consists of two questions; patients are asked to indicate during the past week whether “frequent” bowel movements (or bag changes if stoma bag present) occurred during the day and, separately, during the night. Response options are on a 4-point Likert scale from “not at all” (1 point) to “very much” (4 points). These were summed to calculate the stool frequency subscale score ranging from 2 to 8. By convention, the subscale score totals were linearly transformed to a score from 0 to 100 [33] where higher scores represent a higher level of symptoms (i.e., more frequent bowel movements).

Given our sample size and the fact that the linearly transformed scores were not normally distributed (i.e., 54% had a linearly transformed score of 0), we classified the bowel function outcome in two ways. The first is a binary classification of “normal” (corresponding to “not at all” stool frequency during day and night) and “any impairment” (including all other responses). The second is an ordinal classification of “normal” (corresponding to “not at all” stool frequency during day and night), “minimally impaired” (corresponding to no more than “a little” stool frequency during either day or night) and “considerably impaired” (corresponding to “quite a bit” or “very much” stool frequency during day or night).

### Statistical analysis

For our primary analyses, we assessed the association between the 6-point ACS guideline score for cancer survivors and bowel function impairment modeling bowel function as a binary and ordinal variable. We did the same using the 8-point ACS guideline score for cancer prevention. We used multivariate logistic regression for the binary outcome and cumulative logistic regression for the ordinal outcome to calculate odds ratios (ORs) and 95% confidence intervals (CIs). Models were adjusted for demographic characteristics (age, gender, race/ethnicity) as well as surgical factors that may be potential confounders [time since surgery and primary surgery grouping (Appendix 2)]. Additional adjustment for living arrangement, smoking status, number of comorbidities, specific comorbidities (including inflammatory bowel disease, irritable bowel syndrome, *Helicobacter pylori* infection, diabetes, and hypothyroidism), and alcohol intake (when examining the main score without alcohol) did not change our results, so we omitted these variables from our final models. Where bowel function was treated as an ordinal variable, we compared models with equal and unequal slopes; no meaningful difference was seen between the two approaches for cross-sectional and repeated measures analyses, so we used equal slopes for both. We conducted two sensitivity analyses, one in which we excluded six patients who had received neoadjuvant chemotherapy prior to enrollment and another in which we excluded six patients who had stomas.

For our secondary analyses, we performed repeated measurement analysis to evaluate the association between the baseline 6-point ACS guideline score for cancer survivors and bowel function impairment over time using surveys collected every 6 months up to 36 months after enrollment. Generalized estimating equation (GEE) was used to account for correlation of responses within the same person. We did the same using the 8-point ACS guideline score for cancer prevention.

We conducted an additional analysis to explore whether our results were driven by one or more components of the ACS guideline score for cancer survivors. To do so, we ran multivariate and cumulative logistic regression models including each of the individual components of the ACS guideline instead of the total score. Increments were assigned based on one standard deviation rounded to the nearest whole integer for all variables except physical activity, which was based on the median. In this analysis, we set the values for two people who reported physical activity levels above the 99th percentile to the 99th percentile value to limit the influence of outliers when physical activity was modeled as a continuous variable. There were no outliers in other continuous exposure variables.

Hypothesis tests were two-sided, and the significance threshold was set to a two-sided *p*-value < 0.05. Statistical analyses were performed using SAS version 9.4.

## Results

112 patients (59% female, mean age 59.5 years) met our inclusion criteria. Considering the 6-point ACS guideline score for cancer survivors, 20 (18%) had score of 0–2, 52 (46%) had a score of 3–4, and 40 (36%) had a score of 5–6. Considering the 8-point ACS guideline score for cancer prevention, 16 (14.3%) had a score of 0–3, 41 (36.6%) had a score of 4–5, and 55 (49.1%) had a score of 6–8. There were no statistically significant differences in demographic, clinical (with exception of BMI), disease, or treatment characteristic among patients with different ACS guideline scores for cancer survivors (Table 1) or among patients with different ACS scores for cancer prevention (not shown).

**Table 1:** Characteristics of 112 colon cancer survivors at enrollment in the Lifestyle and Outcomes after Gastrointestinal Cancer (LOGIC) study, by the ACS guideline score for cancer survivors

Characteristic	Total	ACS guideline score for cancer survivors			*p*-value*
		0–2	3–4	5–6	
Total patients, *n* (%)	112	20	52	40	
Demographic characteristics					
Age, mean (SD)	59.5(13.2)	60.6(14.1)	58.9(12.7)	59.8(13.7)	0.87
Gender, *n* (%)					0.85
Female	66(59)	11(55)	32(62)	23(58)	
Male	46(41)	9(45)	20(38)	17(43)	
Race, *n* (%)					0.11
American Indian or Alaska Native	2(2)	0(0)	1(2)	1(3)	
Asian	15(13)	3(15)	2(4)	10(25)	
Black/African American	2(2)	0(0)	2(4)	0(0)	
Native Hawaiian or other Pacific Islander	0 (0)	0 (0)	0 (0)	0 (0)	
White	83(74)	13(65)	43(83)	27(68)	
More than one race	6(5)	2(10)	3(6)	1(3)	
Unknown or not reported	4(4)	2(10)	1(2)	1(3)	
Ethnicity, *n* (%)					0.60
Hispanic or Latino	9(8)	2(10)	5(10)	2(5)	
Not Hispanic or Latino	101(90)	17(85)	46(88)	38(95)	
Unknown or not reported	2(2)	1(5)	1(2)	0(0)	
Living arrangement, *n* (%)					0.12
Alone	14(13)	6(30)	6(12)	2(5)	
With spouse/partner	83(74)	13(65)	37(71)	33(83)	
With other family	10(9)	0(0)	6(12)	4(10)	
Other (e.g., with roommates, etc.)	5(4)	1(5)	3(6)	1(3)	
Clinical characteristics					
Body mass index, mean (SD)	25.7(5.2)	30.0(6.7)	26.6(4.6)	22.5(2.6)	< 0.01
Smoking status, *n* (%)					0.42
Current	4(4)	2(10)	2(4)	0(0)	
Past	36(32)	5(25)	18(35)	13(33)	
No	71(63)	13(65)	32(62)	26(65)	
Missing	1(1)	0(0)	0(0)	1(3)	
Number of comorbidities, *n* (%)					0.69
0	24(21)	3(15)	10(19)	11(28)	
1	32(29)	5(25)	17(33)	10(25)	
2	17(15)	2(10)	9(17)	6(15)	
3 +	39(35)	10(50)	16(31)	13(33)	
Disease characteristics, *n* (%)					
Stage at diagnosis					0.78
Stage I	16(14)	3(15)	10(19)	3(8)	
Stage II	32(29)	7(35)	14(27)	11(28)	
Stage III	51(46)	7(35)	24(46)	20(50)	
Stage IV	8(7)	2(10)	2(4)	4(10)	
Unknown	5(4)	1(5)	2(4)	2(5)	
Metastasis					0.95
Yes	18(16)	3(15)	8(15)	7(18)	
No	94(84)	17(85)	44(85)	33(83)	
Treatment characteristics, *n* (%)					
Primary procedure grouping					0.48
Right/transverse	48(43)	10(50)	23(44)	15(38)	
Left/sigmoid	46(41)	6(30)	21(40)	19(48)	
Low pelvis	9(8)	3(15)	2(4)	4(10)	
Total/subtotal	9(8)	1(5)	6(12)	2(5)	
Time from surgery to enrollment					0.52
Less than 6 months	24(21)	7(35)	10(19)	7(18)	
6 months to 2 years	39(35)	6(30)	17(33)	16(40)	
Greater than 2 years	49(44)	7(35)	25(48)	17(43)	
Chemotherapy					0.50
Neoadjuvant	6(5)	1(5)	1(2)	4(10)	
Adjuvant	61(54)	10(50)	31(60)	20(50)	
None	45(40)	9(45)	20(38)	16(40)	
Health behaviors, mean (SD)					
Physical activity (MET-hours/week)	49.7 (56.6)	10.5 (21.2)	50.5 (60.9)	68.4 (54.0)	< 0.01
Red or processed meat (serving/day)	0.87 (0.64)	1.02 (0.59)	0.96 (0.66)	0.67 (0.60)	0.04
Fruit/vegetable variety (unique fruits/vegetables per month)	24.4 (6.0)	21.5 (5.0)	23.7 (6.0)	26.9 (5.7)	< 0.01
Fruit/vegetable (servings/day)	7.9 (4.5)	7.5 (5.2)	7.2 (4.2)	9.1 (4.5)	0.12
Percent of grains consumed that are whole (%)	58.4 (24.9)	48.0 (28.9)	55.5 (25.2)	67.6 (19.5)	0.01
Alcohol (drinks/day)	0.64 (0.83)	0.48 (1.00)	0.66 (0.81)	0.69 (0.77)	0.63

^*^Chi‐square test was used for categorical variables and ANOVA for continuous variables

^**^Comorbidities that may affect bowel function

### Primary analysis: cross-sectional

The results from the cross-sectional analysis of survey data at enrollment can be seen in Table 2. Adjusted for demographic and surgical characteristics, for every 1-point increase in the 6-point ACS guideline score for cancer survivors, the odds of having any impairment in bowel function decreased by 43% [odds ratio (OR): 0.57; 95% confidence interval (CI): 0.42–0.79; *p* < 0.01) and the odds of being at the next highest level of impairment in bowel function (i.e., minimally impaired vs. normal, considerably impaired vs. minimally impaired) decreased by 46% (OR: 0.54; 95% CI 0.40–0.72; *p* < 0.01).

**Table 2:** Cross-sectional association between adherence to the 6-point ACS guideline score for cancer survivors and bowel function at enrollment among 112 colon cancer survivors

	Bowel function: binary classification				Bowel function: ordinal classification			
	*N* = 112 Events: 47 any impairment				*N* = 112 Events: 30 minimal/17 considerable impairment			
	OR	95% CI		*p*-value	OR	95% CI		*p*-value
		Lower	Upper			Lower	Upper	
6-point ACS guideline score								
Unadjusted	0.64	0.48	0.85	< 0.01	0.61	0.46	0.80	< 0.01
Adjusted model 1*	0.64	0.48	0.84	< 0.01	0.60	0.46	0.78	< 0.01
Adjusted model 2**	0.57	0.42	0.79	< 0.01	0.54	0.40	0.72	< 0.01

^*^Adjusted for age, gender, and race/ethnicity

^**^Adjusted for age, gender, race/ethnicity, time since surgery, and primary surgical procedure grouping

Results were not materially different when the 8-point ACS guideline score for cancer prevention (including alcohol consumption) was used as the exposure. Adjusted for demographic and surgical characteristics, for every 1-point increase in the 6-point ACS guideline score for cancer survivors, the odds of having any impairment in bowel function decreased by 32% [odds ratio (OR): 0.68; 95% confidence interval (CI): 0.52–0.88; *p* < 0.01) and the odds of being at the next highest level of impairment in bowel function (i.e., minimally impaired vs. normal, considerably impaired vs. minimally impaired) decreased by 39% (OR: 0.61; 95% CI 0.47–0.80; *p* < 0.01) (Appendix 3).

Our results were not materially changed when we excluded six patients who received neoadjuvant chemotherapy or the six patients with stomas (data not shown).

### Secondary analysis: repeated measures of bowel function

Results of the repeated measures analysis, in which we examined association between the baseline 6-point ACS guideline score for cancer survivors and bowel function impairment over time using surveys collected every 6 months up to 36 months after enrollment, were similar to the cross-sectional results. The 112 participants included in our study collectively had 384 EORTC QLQ-29 survey responses across 3 years after enrollment; 92 (82%) participants had at least two survey responses. Participants had on average 3.4 EORTC QLQ-29 survey responses (median 3, range 1–7) over this period. No differences in median 6-point ACS guideline score for cancer survivors or demographic (except ethnicity), clinical, disease, or treatment characteristics were observed between participants with 1 vs. 2 or more EORTC QLQ-29 survey responses (Appendix 4).

After adjusting for demographic and surgical characteristics, a 1-point increase in baseline 6-point ACS guideline score for cancer survivors was associated with a 34% decrease in the odds of having any impairment in bowel function (OR: 0.66; 95% CI 0.50–0.88; *p* < 0.01) and a 36% decrease in the odds of being at the next (higher) level of impairment in bowel function (OR: 0.64; 95% CI 0.49–0.84; *p* < 0.01) (Table 3).

**Table 3:** Association between adherence to the 6-point ACS guideline score for cancer survivors at enrollment and bowel function over 36 months after enrollment

	Bowel function: binary classification				Bowel function: ordinal classification			
	*N* = 384 responses Events: 162 any impairment				*N* = 384 responses Events: 104 minimal/58 considerable impairment			
	OR	95% CI		*p*-value	OR	95% CI		*p*-value
		Lower	Upper			Lower	Upper	
6-point ACS guideline score								
Unadjusted	0.72	0.56	0.93	0.01	0.70	0.55	0.91	0.01
Adjusted model 1*	0.71	0.54	0.92	0.01	0.69	0.53	0.89	< 0.01
Adjusted model 2**	0.66	0.50	0.88	< 0.01	0.64	0.49	0.84	< 0.01

^*^Adjusted for age, gender, and race

^**^Adjusted for age, gender, race/ethnicity, time since surgery, and primary surgical procedure grouping

Results were not materially different when the 8-point ACS guideline score for cancer prevention (including alcohol consumption) was used as the exposure. After adjusting for demographic and surgical characteristics, a 1-point increase in baseline 6-point ACS guideline score for cancer survivors was associated with a 30% decrease in the odds of having any impairment in bowel function (OR: 0.70; 95% CI 0.53–0.92; *p* < 0.01) and a 30% decrease in the odds of being at the next (higher) level of impairment in bowel function (OR: 0.70; 95% CI 0.54–0.92; *p* < 0.01) (Appendix 5).

### Secondary analysis: components of the ACS guidelines

Results of the multivariate and cumulative logistic regression models of each individual component of ACS guidelines can be seen in Table 4. Adjusting for age, gender, race, time since surgery, primary surgical procedure type, and all other individual components of ACS guideline score, higher fruit/vegetable variety was associated with better bowel function, with the ordinal model being more sensitive to this difference. Specifically, for every 6 additional types of fruit/vegetables consumed each month, the odds of being at the next highest level of impairment in bowel function decreased by 42% (OR: 0.58; 95% CI 0.35–0.98; *p* = 0.04). For every serving/day increase in red or processed meat, the odds of having any impairment in bowel function increased 2.60-fold (OR: 2.60; 95% CI 1.24–5.49; *p* = 0.01) and the odds of being at the next highest level of impairment in bowel function increased 2.43-fold (OR: 2.43; 95% CI 1.30–4.57; *p* = 0.01). Bowel function impairment was not significantly associated with BMI, total physical activity, servings/day of fruits/vegetables, percent of grains that were whole grains, or alcohol intake in the multivariable models.

**Table 4:** Association between individual ACS guidelines and bowel function at enrollment

	Bowel function: binary classification				Bowel function: ordinal classification			
	*N* = 112 Events: 47 any impairment				*N* = 112 Events: 30 minimal/17 considerable impairment			
	OR	95% CI		*p*-value	OR	95% CI		*p*-value
		Lower	Upper			Lower	Upper	
Body mass index (per 1 SD ≅ 5 units)								
Unadjusted	1.32	0.92	1.90	0.14	1.35	0.95	1.91	0.10
Adjusted model 1*	1.37	0.96	1.95	0.08	1.39	1.00	1.94	0.05
Adjusted model 2**	1.45	0.99	2.14	0.06	1.49	1.05	2.11	0.03
Adjusted model 3***	1.30	0.84	2.01	0.25	1.33	0.90	1.98	0.15
Physical activity (per 30 MET-h/week)								
Unadjusted	0.76	0.54	1.07	0.12	0.74	0.52	1.06	0.10
Adjusted model 1*	0.75	0.54	1.04	0.08	0.69	0.47	1.00	0.05
Adjusted model 2**	0.78	0.56	1.09	0.15	0.72	0.50	1.04	0.08
Adjusted model 3***	0.87	0.64	1.18	0.36	0.82	0.59	1.13	0.23
Red or processed meat (per 1 SD ≅ serving/day)								
Unadjusted	1.79	1.00	3.19	0.05	1.71	1.02	2.88	0.04
Adjusted model 1*	1.99	1.08	3.67	0.03	1.99	1.16	3.42	0.01
Adjusted model 2**	2.35	1.17	4.70	0.02	2.21	1.25	3.92	0.01
Adjusted model 3***	2.60	1.24	5.49	0.01	2.43	1.30	4.57	0.01
Fruit/vegetable variety (per 1 SD ≅ 6 units)								
Unadjusted	0.84	0.57	1.23	0.37	0.75	0.52	1.09	0.14
Adjusted model 1*	0.85	0.57	1.26	0.42	0.75	0.51	1.11	0.16
Adjusted model 2**	0.76	0.47	1.21	0.25	0.63	0.39	0.99	0.05
Adjusted model 3***	0.75	0.45	1.25	0.27	0.58	0.35	0.98	0.04
Fruits/vegetables (per 1 SD ≅ 5 serving/day)								
Unadjusted	0.84	0.56	1.26	0.40	0.86	0.57	1.28	0.45
Adjusted model 1*	0.84	0.56	1.25	0.39	0.86	0.58	1.28	0.47
Adjusted model 2**	0.84	0.53	1.30	0.43	0.87	0.57	1.34	0.54
Adjusted model 3***	0.89	0.47	1.71	0.73	1.08	0.60	1.93	0.80
Whole grain percentage (per 1 SD ≅ 20 percent)								
Unadjusted	0.97	0.71	1.31	0.83	0.98	0.71	1.35	0.88
Adjusted model 1*	0.94	0.68	1.30	0.73	0.98	0.70	1.36	0.89
Adjusted model 2**	0.91	0.65	1.27	0.57	0.95	0.67	1.34	0.77
Adjusted model 3***	0.98	0.67	1.41	0.89	1.03	0.70	1.50	0.89
Alcohol use (per 1 SD ≅ 1 drink/day)								
Unadjusted	0.76	0.47	1.22	0.25	0.74	0.46	1.20	0.23
Adjusted model 1*	0.81	0.50	1.32	0.40	0.80	0.50	1.31	0.38
Adjusted model 2**	0.73	0.44	1.19	0.21	0.74	0.45	1.21	0.22
Adjusted model 3***	0.71	0.42	1.19	0.19	0.74	0.43	1.28	0.28

^*^Adjusted for age, gender, and race/ethnicity

^**^Adjusted for age, gender, race/ethnicity, time since surgery, and primary surgical procedure grouping

^***^Adjusted for age, gender, race/ethnicity, time since surgery, primary surgical procedure grouping, and all other individual components of ACS score

## Discussion

In this study of colon cancer survivors, we sought to determine whether adherence to the ACS guidelines for body size, nutrition, and physical activity was associated with less bowel function impairment, measured by stool frequency. While bowel function is a widely studied topic in rectal cancer [14, 16, 34, 34–36], few studies have investigated patient-driven factors that affect bowel function after surgery for colon cancer. Our results from cross-sectional and repeated measure models consistently demonstrated that colon cancer survivors who more closely follow the ACS nutrition and physical activity guidelines had decreased odds of bowel function impairment. This finding suggests the value of interventions that support health behavior modification as part of colon cancer survivorship care.

Health behaviors, including nutrition and physical activity, have been shown to have extensive benefits for the treatment [37–42] and prevention [25, 43–45] of multiple disease processes. In patients diagnosed with cancer, they may confer benefits in recurrence and survival [23, 24, 46, 47] as well as improve wellbeing and QoL [48–50]. Adherence to the ACS Nutrition and Physical Activity Guidelines has been correlated with higher QoL for colorectal cancer survivors [51] and longer survival among stage III colon cancer patients [11]. Findings from our study reinforce the value of adherence to the ACS guidelines in colon cancer survivorship by identifying another benefit (i.e., improved bowel function), expanding the current literature base to further support health behavior modification in this group.

Notably, while health behavior modification may not always confer noticeable changes in symptoms [52–54], in our population, greater adherence to the ACS nutrition and physical activity guidelines was associated with better bowel function. There is a complex and intricate balance between instant gratification and delayed rewards in human behavior [55–57]. A diet high in fat, sugar, carbohydrates, and meat results in instant gratification [58] whereas benefits of fruits and vegetables and physical activity are delayed [58]. Prior work has shown that immediate rewards are valued higher than larger rewards that are delayed [59], which may explain the difficulty in making behavior changes that favor long-term health over instant gratification. Though further exploration is needed to confirm causality and determine the time-course of the effect, our study suggests the possibility of perceptible change (i.e., improved bowel function) in the near-term resulting from health behavior modification *in addition to* the well-known, widely accepted long-term health benefits that may not be physically felt. This may offer salient motivation to colon cancer survivors for implementing behavioral modifications.

Importantly, our study has actionable implications for practitioners who care for colon cancer survivors. Our findings revealed two modifiable dietary factors that may be particularly salient for bowel function: reducing consumption of red or processed meat and increasing the variety or number of unique fruits and vegetables consumed. Consumption of red and processed meat before diagnosis has been implicated (albeit inconclusively) in the development of colorectal cancer [60, 61]. Moreover, the beneficial role of fruits and vegetables has been broadly accepted [62], though their benefit against development of colon cancer remains unclear [63]. Our study offers evidence that red and processed meat may be independently and strongly associated with worse bowel function, while a greater variety of fruit and vegetable consumption is associated with better bowel function among colon cancer survivors after adjusting for known risk factors. Importantly, these data offer practitioners evidence-based guidance on where to focus colon cancer survivorship recommendations. Although the notions that consumption of red and processed meats carries risk while consumption of fruits and vegetables confers benefit are not inherently novel nor surprising, these findings establish an emerging evidence-base that may be shared with patients to further support behavior change and ultimately improve bowel function and QoL.

One potentially surprising finding from our study is the lack of significant association between bowel function and physical activity. Notably, prior literature has demonstrated that increased physical activity is associated with a reduction in the prevalence of constipation [64], shorter colon transit time [65], and increased stool frequency [66] in other settings. However, to our knowledge, association between physical activity and bowel function, specifically stool frequency, in colon cancer survivors has not been examined. It is conceivable that post-treatment changes in colonic function among this population respond to the effects of physical activity differently than other groups. It is also possible that we were unable to detect an association due to the limited variability in physical activity in our sample; physical activity levels were generally high in our study population. This finding warrants further investigation.

## Limitations

While our findings begin to offer insights into the role of health behaviors in colon cancer survivors’ bowel function, they should be viewed in the context of several limitations. First, we considered stool frequency as a proxy for bowel function in the absence of a validated bowel function score for colon cancer survivors [19]. Although stool frequency has been used as a clinical surrogate for gut transit [67] and is the most frequently used measure of bowel function for this group [19], this measure does not capture the full spectrum of bowel impairment, which includes constipation, urgency, and incontinence, among others. Future efforts should be directed toward assessing the association between health behaviors and these individual measures, as well as developing a comprehensive bowel function score for colon cancer survivors. Second, causality is impossible to ascertain from any observational study. However, our prospective outcome data at multiple time points suggest a temporal association. Further study with additional time-points on both the health behaviors and stool frequency is needed. Third, not all patients enrolled in our study for 6 + months had stool frequency data at two or more time points. This may introduce selection bias in our repeated measures analysis from loss to follow-up. However, there were no differences in baseline ACS guideline score or clinical, disease, or treatment characteristics between participants with one vs. 2 or more EORTC QLQ-29 surveys, suggesting this bias was minimal. Fourth, our cohort had an over-representation of patients identifying as white and Asian race and those with high adherence to the ACS guidelines [11, 68], highlighting the need for diversification of future enrollment. Finally, the average daily consumption of servings of fruits and vegetables and number of alcoholic beverages per day of our overall cohort was in line with the ACS guidelines and these measures were not significantly different across individuals with different overall ACS scores. These characteristics of our cohort may have made an association with bowel function difficult to detect, further underscoring the need for diversification of future enrollment. Nonetheless, our findings provide a starting point for evidence-based guidance to inform shared decision-making with colon cancer survivors around modifiable health behaviors.

## Conclusion

Our findings suggest that colon cancer survivors who more closely follow the ACS nutrition and physical activity guidelines have lower odds of bowel function impairment, as determined by stool frequency. This finding supports the value of health behavior interventions as part of survivorship care for these individuals. Further study is needed to understand the longitudinal impact of health behaviors on bowel function in this group, as well as in more racially/ethnically and socioeconomically diverse populations of cancer survivors.

## Supplementary Information

Below is the link to the electronic supplementary material.

Supplementary file1 (DOCX 34 KB)
