# Supplementary material for: Association between adherence to the American Cancer Society Nutrition and Physical Activity Guidelines and stool frequency among colon cancer survivors: a cohort study
Source: J Cancer Surviv. Author manuscript; Available in PMC 2023 Jul 12. (PMC10209241; doi:10.1007/s11764-022-01288-8)
Supplement: Supplementary Material [file NIHMS1893611-supplement-Supplementary_Material.docx]

**Appendix 1**. Description of ACS Nutrition and Physical Activity Guidelines for Cancer Survivors and Prevention Score

| **Recommendation** | **Description** | **Possible Points** |
| --- | --- | --- |
| **“Maintain a healthy weight throughout life”** | | **0-2** |
|  | *BMI ≥30 kg/m2* | *0* |
|  | *BMI 25 - 30 kg/m2* | *1* |
|  | *BMI 18.5 - <25kg/m2* | *2* |
|  |  |  |
| **“Adopt a physically active lifestyle”** |  | **0-2** |
|  | *<8.75 MET-h/wk* | *0* |
|  | *8.75 - <17.5 MET-h/wk* | *1* |
|  | *≥17.5 MET-h/wk* | *2* |
|  |  |  |
| **“Consume a healthy diet with emphasis on plant sources”** |  | **0-2^1^** |
| - *Limit consumption of processed meat and red meats* | *Based on quartile distribution of servings consumed/week (lowest quartile = 3)* | *0-3* |
| - *Eat 5+servings of a variety of vegetables and fruits each day* | *<5 servings/day* | *0* |
|  | *≥5 servings/day* | *1* |
|  | *2nd or 3rd tertile of unique fruits and vegetables consumed/month* | *2-3* |
| - *Choose whole grains in preference to processed (refined) grains* | *Based on quartile distribution (highest quartile = 3)* | *0-3* |
|  |  |  |
| **“If you drink, limit consumption to 1 drink/day for women and 2 drinks/day for men”** | | **0-2** |
|  | *>1 drink/day for women, >2 drinks/day for men* | *0* |
|  | *Non-drinker* | *1* |
|  | *>0–1 drink/day for women, >0–2 drinks/day for men* | *2* |
|  |  |  |

BMI = Body Mass Index; MET = metabolic equivalent task value

**^1^**Dietary factors (including fruits and vegetables, whole grains, and red and processed meat) were summed; individuals with 0-2 diet points were given 0 points, 3-6 points were given 1 point, and 7-9 points were given 2 points. F

**Appendix 2**. Primary Surgery Grouping Definitions

| **Grouping** | **Primary Surgery** |
| --- | --- |
| **Right/Transverse** | |
|  | Right hemicolectomy |
|  | Extended right colectomy |
|  | Transverse hemicolectomy |
| **Left/Sigmoid** |  |
|  | Left hemicolectomy |
|  | Extended left colectomy |
|  | Sigmoid colectomy |
| **Low pelvis** |  |
|  | Low anterior resection |
|  | Low anterior resection with diverting loop ileostomy |
| **Total/Subtotal** |  |
|  | Total proctocolectomy with end ileostomy |
|  | Total proctocolectomy with ileal pouch-anal anastomosis |
|  | Total colectomy with ileorectal anastomosis |
|  | Total abdominal colectomy with end ileostomy |
|  | Subtotal colectomy with ileosigmoid anastomosis |

**Appendix 3**. Cross-sectional association between adherence to the 8-point ACS Guidelines Score for Cancer Prevention and bowel function at enrollment among 112 colon cancer survivors

|  | **Bowel Function: Binary Classification** | | | |  |  | | **Bowel Function: Ordinal Classification** | | | | | | | |  |
| --- | --- | --- | --- | --- | --- | --- | --- | --- | --- | --- | --- | --- | --- | --- | --- | --- |
|  | **N=112  Events: 47 any impairment** | | | |  |  | | **N=112  Events: 30 minimal, 17 considerable impairment** | | | | | | | | |
|  | **OR** | **95% CI** |  | **p-value** | | |  | | **OR** | | **95% CI** | |  | | **p-value** | |
|  |  | **Lower** | **Upper** |  |  |  |  | |  |  | **Lower** | | **Upper** | |  |  |
| **8-point ACS Guidelines Score** |  |  |  |  | | |  | |  | |  | |  | |  | |
| Unadjusted | 0.73 | 0.57 | 0.93 | 0.01 | | |  | | 0.69 | | 0.54 | | 0.88 | | <0.01 | |
| Adjusted model 1* | 0.73 | 0.57 | 0.94 | 0.01 | | |  | | 0.68 | | 0.53 | | 0.87 | | <0.01 | |
| Adjusted model 2** | 0.68 | 0.52 | 0.88 | <0.01 | | |  | | 0.61 | | 0.47 | | 0.80 | | <0.01 | |
| * Adjusted for age, gender, and race/ethnicity | | | | | |  | |  | | | |  | |  | |  |
| ** Adjusted for age, gender, race/ethnicity, time since surgery, and primary surgical procedure group | | | | | | | | | |  | |  | |  | |  |

**Appendix 4**. Participant Characteristics by Number of EORTC QLQ-29 Surveys Completed in 3-Year Period

| **Characteristic** | **All** | **EORTC QLQ-29 Surveys Completed in 3-Year Period** | | |
| --- | --- | --- | --- | --- |
|  |  | **1** | **2+** | **p-value*** |
| **Total Patients, n (%)** | 112 | 20 | 92 |  |
| **Demographic Characteristics** |  |  |  |  |
| **Age, Mean (SD)** | 59.5 (13.2) | 62.0 (16.2) | 59.0 (12.5) | 0.37 |
| **Gender, n (%)** |  |  |  | 0.54 |
| Female | 66 (59) | 13 (65) | 53 (58) |  |
| Male | 46 (41) | 7 (35) | 39 (42) |  |
| **Race, n (%)** |  |  |  | 0.41 |
| American Indian or Alaska Native | 2 (2) | 0 (0) | 2 (2) |  |
| Asian | 15 (13) | 3 (15) | 12 (13) |  |
| Black / African American | 2 (2) | 0 (0) | 2 (2) |  |
| Native Hawaiian or other Pacific Islander | 0 (0) | 0 (0) | 0 (0) |  |
| White | 83 (74) | 15 (75) | 68 (74) |  |
| More than one race | 6 (5) | 0 (0) | 6 (7) |  |
| Unknown or not reported | 4 (4) | 2 (10) | 2 (2) |  |
| **Ethnicity, n (%)** |  |  |  | <0.01 |
| Hispanic or Latino | 9 (8) | 0 (0) | 9 (10) |  |
| Not Hispanic or Latino | 101 (90) | 18 (90) | 83 (90) |  |
| Unknown or not reported | 2 (2) | 2 (10) | 0 (0) |  |
| **Living Arrangement, n (%)** |  |  |  | 0.21 |
| Alone | 14 (13) | 4 (20) | 10 (11) |  |
| With spouse/partner | 83 (74) | 16 (80) | 67 (73) |  |
| With other family | 10 (9) | 0 (0) | 10 (11) |  |
| Other (e.g., with roommates, etc.) | 5 (4) | 0 (0) | 5 (5) |  |
| **Clinical Characteristics** |  |  |  |  |
| **Body Mass Index, Mean (SD)** | 25.7 (5.2) | 26.4 (5.8) | 25.6 (5.1) | 0.54 |
| **Smoking Status, n (%)** |  |  |  | 0.76 |
| Current | 4 (4) | 0 (0) | 4 (4) |  |
| Past | 36 (32) | 7 (35) | 29 (32) |  |
| No | 71 (63) | 13 (65) | 58 (63) |  |
| Missing | 1 (1) | 0 (0) | 1 (1) |  |
| **Number of Comorbidities, n (%)** |  |  |  | 0.21 |
| 0 | 24 (21) | 1 (5) | 23 (25) |  |
| 1 | 32 (29) | 8 (40) | 24 (26) |  |
| 2 | 17 (15) | 4 (20) | 13 (14) |  |
| 3+ | 39 (35) | 7 (35) | 32 (35) |  |
| **Disease Characteristics, n (%)** |  |  |  |  |
| **Stage at Diagnosis** |  |  |  | 0.48 |
| Stage I | 16 (14) | 4 (20) | 12 (13) |  |
| Stage II | 32 (29) | 8 (40) | 24 (26) |  |
| Stage III | 51 (46) | 7 (35) | 44 (48) |  |
| Stage IV | 8 (7) | 1 (5) | 7 (8) |  |
| Unknown | 5 (4) | 0 (0) | 5 (5) |  |
| **Metastasis** |  |  |  | 0.89 |
| Yes | 18 (16) | 3 (15) | 15 (16) |  |
| No | 94 (84) | 17 (85) | 77 (84) |  |
| **Treatment Characteristics, n (%)** |  |  |  |  |
| **Primary Surgery (Grouped)** |  |  |  | 0.33 |
| Right/Transverse | 48 (43) | 10 (50) | 38 (41) |  |
| Left/Sigmoid | 46 (41) | 5 (25) | 41 (45) |  |
| Low Pelvis | 9 (8) | 2 (10) | 7 (8) |  |
| Total/Subtotal | 9 (8) | 3 (15) | 6 (7) |  |
| **Time from Surgery to Study Enrollment** |  |  |  | 0.12 |
| Less than 6 months | 24 (21) | 7 (35) | 17 (19) |  |
| 6 months to 2 years | 39 (35) | 8 (40) | 31 (34) |  |
| Greater than 2 years | 49 (44) | 5 (25) | 44 (48) |  |
| **Chemotherapy** |  |  |  | 0.32 |
| Neoadjuvant | 6 (5) | 1 (5) | 5 (5) |  |
| Adjuvant | 61 (54) | 8 (40) | 53 (58) |  |
| None | 45 (40) | 11 (55) | 34 (37) |  |
| **Lifestyle Factors, mean (SD)** |  |  |  |  |
| **Physical activity (MET-hours/week)** | 49.7 (56.6) | 50.0 (60.7) | 49.7 (56.0) | 0.98 |
| **Red or Processed Meat (serving/day)** | 0.87 (0.64) | 0.80 (0.56) | 0.88 (0.66) | 0.59 |
| **Fruit/Vegetable Variety (unique fruits/vegetables per month)** | 24.4 (6.0) | 23.7 (5.9) | 24.6 (6.0) | 0.51 |
| **Fruit/Vegetable (servings per day)** | 7.9 (4.5) | 7.9 (4.5) | 7.9 (4.5) | 1.00 |
| **Percent of grains consumed that are whole** | 58.4 (24.9) | 55.2 (34.2) | 59.2 (22.6) | 0.52 |
| **Alcohol (drinks/day)** | 0.64 (0.83) | 0.53 (0.65) | 0.66 (0.86) | 0.52 |
| * Chi‐square test was used for categorical variables and ANOVA for continuous variables | | |  |  |

**Appendix 5**. Association between adherence to the 8-point ACS Guidelines Score for Cancer Prevention at enrollment and Bowel Function over 36-months after enrollment

|  | **Bowel Function: Binary Classification** | | | |  | **Bowel Function: Ordinal Classification** | | |  |
| --- | --- | --- | --- | --- | --- | --- | --- | --- | --- |
|  | **N=384 Responses  Events: 162 any impairment** | | | |  | **N=384 Responses Events: 104 minimal/58 considerable impairment** | | | |
|  | **OR** | **95% CI** |  | **p-value** |  | **OR** | **95% CI** |  | **p-value** |
|  |  | **Lower** | **Upper** |  |  |  | **Lower** | **Upper** |  |
| **8-point ACS Guidelines Score** |  |  |  |  |  |  |  |  |  |
| Unadjusted | 0.74 | 0.58 | 0.94 | 0.01 |  | 0.75 | 0.58 | 0.95 | 0.02 |
| Adjusted Model 1* | 0.73 | 0.57 | 0.93 | 0.01 |  | 0.74 | 0.58 | 0.95 | 0.02 |
| Adjusted Model 2** | 0.70 | 0.53 | 0.92 | <0.01 |  | 0.70 | 0.54 | 0.92 | <0.01 |
| * Adjusted for age, gender, and race | |  |  |  |  |  |  |  |  |
| ** Adjusted for age, gender, race, time since surgery, and primary procedure grouping | | | | |  |  |  |  |  |
